# Single-Stage Repair and Reconstruction of Multiligament Injury With Bicondylar Tibial Plateau Fracture Following Traumatic Posterolateral Knee Dislocation: A Case Report

**DOI:** 10.7759/cureus.98659

**Published:** 2025-12-07

**Authors:** Christian Roberti, Savannah R Chapman, John Paul Lemchak, Margaret Mueller

**Affiliations:** 1 Medicine, Lake Erie College of Osteopathic Medicine, Bradenton, USA; 2 Emergency Medicine, Sturdy Memorial Hospital, Attleboro, USA

**Keywords:** knee dislocation, lcl, mcl tear, multiligament injury, multiligament knee injuries, patellar tendon injury

## Abstract

Knee dislocations (KDs) accompanied by bicondylar tibial plateau fractures and multiligament injuries are uncommon and present significant technical challenges, particularly in patients at high thrombotic risk where tourniquet use is contraindicated. We present the case of a 30-year-old male who sustained a traumatic posterolateral KD with a bicondylar tibial plateau fracture, meniscal and capsular disruption, and patellar tendon and multiligament injury following a high-velocity motor vehicle accident. Due to bilateral lower-extremity deep venous thromboses, surgery was performed without a tourniquet using a single-stage combined arthroscopic and open approach. The medial meniscus, medial collateral ligament, joint capsule, and patellar tendon were repaired with suture anchors, while the lateral collateral ligament and popliteus tendon were reconstructed using an allograft. This approach achieved anatomic realignment, restored knee stability, and preserved peroneal nerve function. At three months, the patient demonstrated a full range of motion and returned to normal activity. This case highlights that complex KDs with bicondylar tibial plateau fractures can be safely and effectively managed in a single stage without a tourniquet. The described technique provides practical strategies for surgeons managing similar high-risk patients and contributes to the development of more standardized operative protocols for complex multiligament knee injuries.

## Introduction

Knee dislocation (KD) with concomitant bicondylar tibial plateau fracture and multiligament injury is rare, and intraoperative algorithms are poorly standardized, particularly when thrombotic risk precludes tourniquet use [1]. KDs account for approximately 0.2% of orthopedic trauma and may be accompanied by serious complications such as thrombosis or disruption of the popliteal artery and vein. They are most commonly caused by high-impact trauma to the knee, including motor vehicle accidents and sports injuries [2]. These injuries pose significant clinical challenges because the combination of ligamentous and osseous disruption increases the risk of neurovascular compromise and complicates surgical planning.

Diagnosis is often difficult because these injuries may spontaneously reduce, and vascular injury may not be immediately suspected. Additionally, plain radiographs may be negative for acute abnormalities, including tibial plateau fractures [3]. Given these diagnostic challenges, thorough vascular assessment is essential, typically using ankle-brachial index (ABI), Doppler US, or CT angiography, with angiography often preferred [4]. After vascular integrity is confirmed and the joint is reduced, MRI is used to evaluate ligamentous injury. The Schenck classification system, which categorizes KDs based on ligamentous and osseous injury patterns, helps guide prognosis and surgical planning, grading injuries from KD-I to KD-V with subcategories. Treatment decisions are generally based on clinician judgment, as there is no universally accepted protocol; however, KD-I and KD-II injuries are typically managed conservatively [3]. These principles frame the surgical challenges and decision-making required in managing complex KD-V injuries such as the present case.

This case is particularly novel because it involves a single-stage, combined open and arthroscopic repair performed without a tourniquet due to bilateral deep vein thromboses (DVTs), a scenario not well described in existing literature. When surgery is indicated, there remains no clear consensus regarding the optimal approach, specifically whether to perform reconstruction using autograft or allograft, or whether to pursue a single-stage versus staged procedure. Autograft reconstruction is generally recommended within three weeks, though debate persists about whether a staged or single-stage strategy is superior [5]. Careful preoperative planning is therefore crucial to restore joint stability while minimizing intraoperative risk.

The purpose of this report is to describe the management of a complex KD-V injury with concomitant bicondylar tibial plateau fracture, multiligament disruption, and tendon injury in a patient with bilateral DVTs, which contraindicated tourniquet use. We highlight technical modifications, including combined arthroscopic and open repair of meniscal, capsular, ligamentous, and tendinous structures, and strategies for achieving global stability, emphasizing the case’s novelty and clinical relevance. By presenting this case, we aim to provide practical considerations for surgeons managing similarly high-risk patients and to contribute toward developing more standardized approaches for complex KDs complicated by osseous injury and vascular risk factors.

## Case presentation

**Figure 1:**
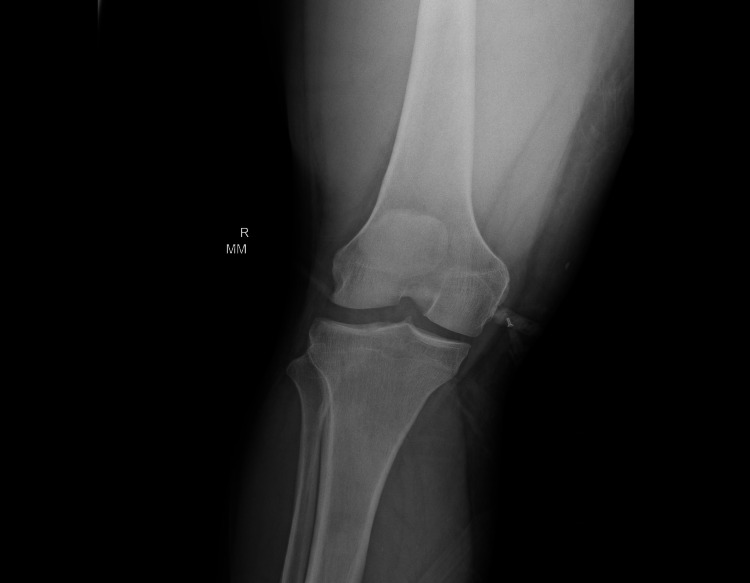
Right knee AP radiograph

A 30-year-old male presented to the emergency department following a high-velocity motor vehicle accident with an obvious deformity of the right knee consistent with a posterolateral dislocation. As an emergency measure to preserve vascularity, closed reduction was performed using the straightened-leg technique, which was uncomplicated. The knee was then stabilized with an external fixator to protect vascular structures and maintain joint alignment. As shown in Figure 1, the initial AP radiograph of the right knee was negative for acute osseous abnormalities.

**Figure 2:**
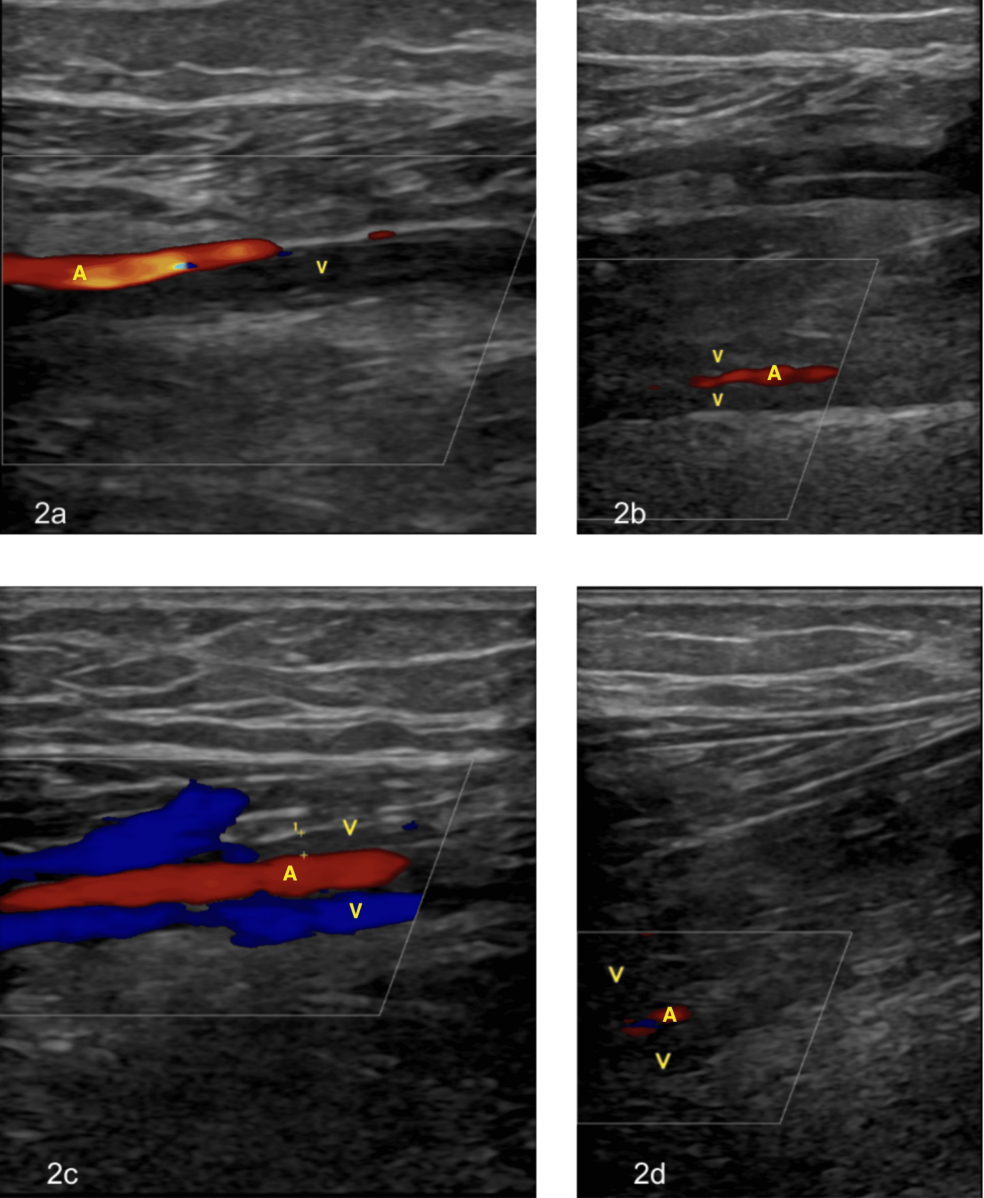
Doppler US demonstrating occlusive thrombus formation in the bilateral posterior tibial and peroneal veins (a) Occlusive thrombus in the right posterior tibial vein with Doppler signal confirming arterial flow. (b) Occlusive thrombus in the right peroneal veins with a Doppler signal confirming arterial flow. (c) Occlusive thrombus in one of the paired posterior tibial veins with preserved arterial flow and flow in the adjacent paired vein. (d) Occlusive thrombus in the left peroneal veins with a Doppler signal confirming arterial flow. A, artery; V, vein

Venous Doppler US demonstrated bilateral DVTs involving the peroneal and posterior tibial veins (Figure 2). An inferior vena cava filter was placed prior to surgery. Due to the patient’s thrombotic risk, a tourniquet was contraindicated for the entire procedure.

**Figure 3:**
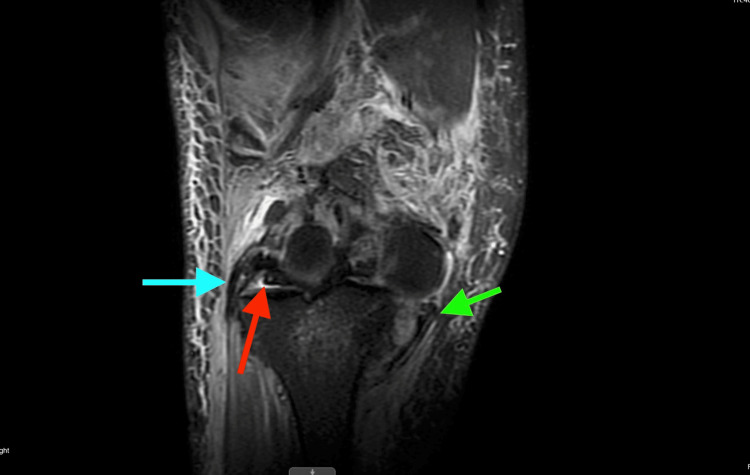
MRI demonstrating extensive ligamentous, osseous, capsular, and meniscal injury with malalignment of the tibia relative to the femur The green arrow indicates the MCL tear, the blue arrow indicates the LCL tear, and the red arrow indicates meniscal disruption. LCL, lateral collateral ligament; MCL, medial collateral ligament

**Figure 4:**
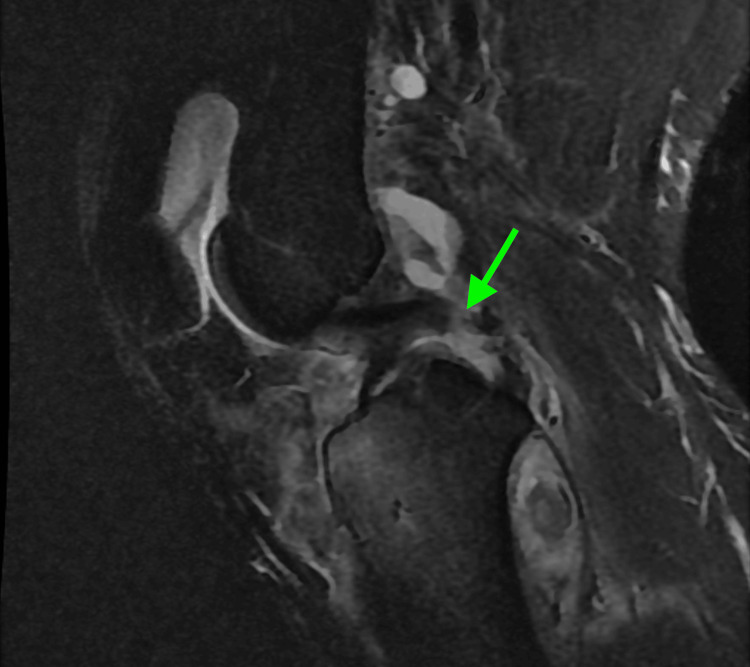
MRI demonstrating mid-substance PCL tear The green arrow indicates the PCL tear. PCL, posterior cruciate ligament

**Figure 5:**
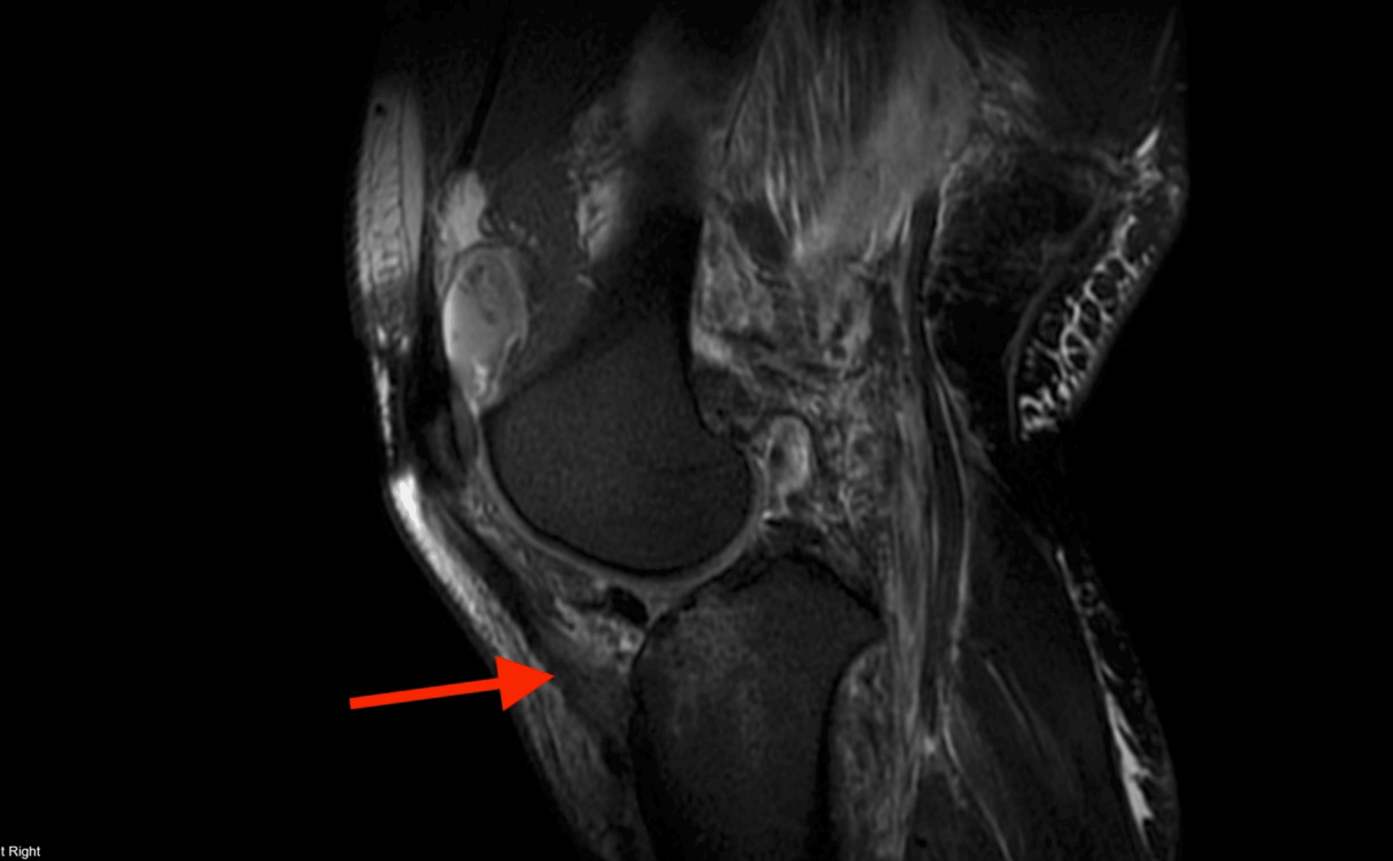
MRI demonstrating patellar tendon disruption near its tibial attachment The red arrow indicates the patellar tendon tear.

**Figure 6:**
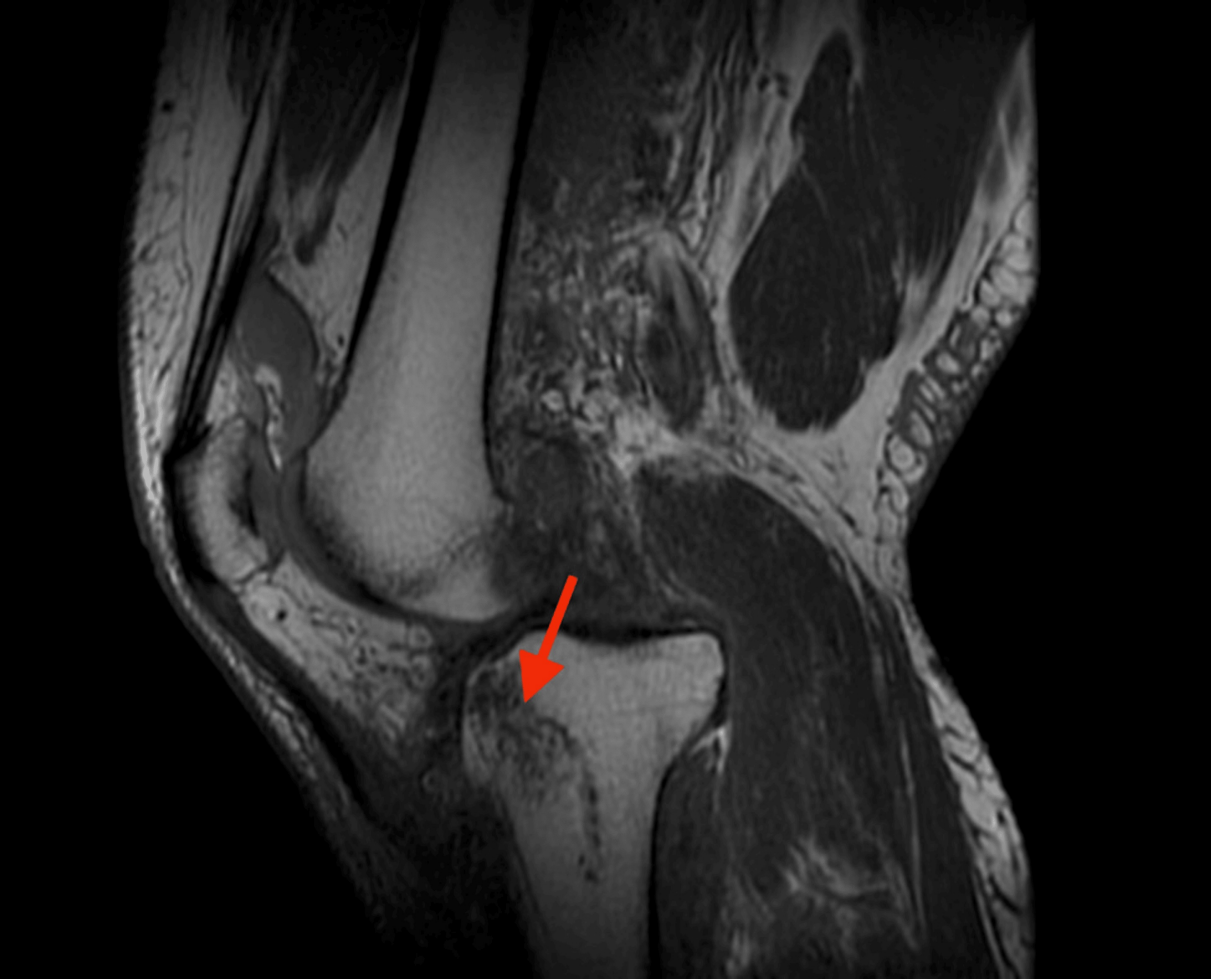
MRI demonstrating anterior tibial plateau fracture The red arrow indicates the tibial plateau fracture.

MRI revealed extensive injury, including tears of the lateral and medial menisci; disruption of the lateral collateral ligament (LCL), medial collateral ligament (MCL), and posterior cruciate ligament (PCL); tears of both gastrocnemius tendon origins; disruption of the lateral patellofemoral ligament and popliteus tendon; a bicondylar tibial plateau fracture; a partial medial patellofemoral ligament tear; myositis and significant soft-tissue swelling; hematoma formation adjacent to the tibial tubercle and within the lateral venous structures; and anterior-medial displacement of the femur relative to the tibia (Figure 3, Figure 4, Figure 5, Figure 6).

Under general anesthesia, the external fixator previously applied to the right lower extremity was removed without complication. Examination under anesthesia confirmed gross varus and valgus instability with minimal AP translation. The leg was then prepped and draped in the standard sterile fashion.

An inferomedial arthroscopic portal was established, revealing extensive hematoma and synovitis that limited visualization. A meticulous synovectomy and debridement were performed in all three compartments to clear the joint and achieve hemostasis. The medial compartment demonstrated complete meniscocapsular avulsion from the medial tibial plateau with subluxation of the tibiofemoral joint. The lateral compartment showed no meniscal or chondral damage. The anterior cruciate ligament and femoral PCL origin appeared intact, although the PCL tibial attachment was disrupted. Given the posterior capsular tear and gastrocnemius muscle involvement, the posterior compartment was not explored arthroscopically to avoid exacerbating swelling and the risk of compartment syndrome. Due to the extent of medial disruption, the procedure was converted to an open approach.

A medial incision extending from the medial epicondyle to the pes anserine area revealed complete disruption of the MCL complex, joint capsule, and patellar tendon insertion. The medial meniscus body was repaired using polydioxanone 0 sutures to restore its contour. Two all-suture anchors were inserted into the proximal tibial plateau to secure the meniscocapsular junction, followed by repair of the anteromedial capsule using an additional anchor at the pes anserine area. The patellar tendon was reattached to the tibial tubercle using another anchor, ensuring solid fixation, and the MCL was reattached to its femoral origin using an all-suture anchor, restoring medial stability. Arthroscopic reassessment after repair confirmed anatomic alignment and elimination of knee subluxation.

Attention was then turned to the lateral side. A lateral incision from the lateral epicondyle to Gerdy’s tubercle was made, and the common peroneal nerve was carefully identified, released, and tagged for protection. A 7-mm presutured allograft was prepared for combined LCL and popliteus tendon reconstruction. A guide pin was placed from the anterolateral fibular head to the posteromedial cortex, reamed bicortically, and the graft was passed through the tunnel and secured with a biocomposite interference screw. Two parallel femoral tunnels were drilled proximal-posterior and distal-deep to the lateral epicondyle. The graft limbs were passed under the iliotibial band, through the femoral tunnels, and tensioned appropriately after cycling the knee 20 times to remove slack. Fixation was completed with interference screws inserted at 20° of flexion under valgus stress for the LCL limb and at 60° of flexion for the popliteus limb.

Following fixation, the knee demonstrated a negative Lachman test, a negative posterior sag sign, and no residual varus or valgus instability. The patellofemoral joint tracked normally, and fluoroscopic imaging confirmed anatomic alignment without evidence of redislocation or subluxation. The iliotibial band, subcutaneous tissue, and skin were closed in layers. The patient emerged from anesthesia with intact peroneal nerve function and normal dorsiflexion and plantarflexion.

At three months postoperatively, the patient remained compliant with physical therapy, demonstrated a full range of motion, and was able to ambulate without assistance. He reported minimal pain with valgus and varus stress and only mild discomfort during flexion or extension.

## Discussion

KD-V KDs involve both fracture and ligamentous injury, making them among the most complex orthopedic injuries to manage. Schenck et al. emphasize that much of the existing literature lacks sufficient detail when reporting these cases, which continues to hinder the development of standardized management protocols [6]. Although numerous studies describe lower-grade KDs, reports specifically addressing grade V injuries remain limited. Table 1 summarizes the Schenck and Wascher classification system for KDs and the associated injury patterns [3].

**Table 1:** Schenck and Wascher classifications of KDs KD, knee dislocation Data adapted from McKee et al. (2014) [3]

Group	Subgroup	Definition
KD-I		Single cruciate only
KD-II		Bicruciate disruption only (rare)
KD-III		Bicruciate and posteromedial or posterolateral disruption (common)
KD-IV		Bicruciate and posteromedial and posterolateral disruption
KD-V		Dislocation with associated fracture
	KD-V1	Single cruciate only
	KD-V2	Bicruciate disruption only
	KD-V3M	Bicruciate and posteromedial disruption
	KD-V3L	Bicruciate and posterolateral disruption
	KD-V4	Bicruciate and posteromedial and posterolateral disruption
	C	Indicates associated arterial injury when suffixed to the main group
	N	Indicates associated neural injury when suffixed to the main group

Most KD-V cases in the literature are managed in a staged fashion, with initial external fixation followed by delayed ligament reconstruction, or with selective fixation of the fracture and later ligamentous repair. This patient underwent initial external fixation after closed reduction of the knee, followed by reconstruction and repair four weeks post-injury. The tibial plateau fracture was not surgically repaired, as it was nondisplaced and therefore amenable to conservative management. Similarly, the PCL tear was left unrepaired to minimize additional soft-tissue trauma and reduce operative duration, given the contraindication to tourniquet use. This staged approach is often favorable for severe dislocations, as tissues with potential for natural healing are given time to recover, leaving only irreparable structures to be surgically addressed. Furthermore, there is evidence of improved joint mobility and stability with this strategy, as reflected in our patient’s satisfactory functional results three months postoperatively [7].

The clinical and surgical sequence in this case highlights several important considerations. Plain radiographs did not reveal the patient’s knee abnormalities after reduction, which could have resulted in misdiagnosis and long-term vascular complications had MRI not confirmed the extent of injury. Additionally, we chose to repair rather than reconstruct the MCL and patellar tendon despite the procedure occurring beyond the three-week window when reconstruction is generally recommended, according to Ng et al. [7].

Due to the extent of posterolateral tissue disruption, reconstruction of the LCL and popliteus tendon with an allograft was selected over repair. Another key technical consideration was avoiding posterior arthroscopy to minimize compartment pressure in the setting of posterior capsular and gastrocnemius injury, as postoperative compartment syndrome could threaten limb survival [8].

Vascular compromise occurs in up to 64% of KDs [9]. For this reason, ABI measurement, Doppler US, and CT angiography remain essential first-line investigations [4]. However, most published case reports emphasize arterial disruption, while venous thrombosis and the implications of tourniquet use in the setting of venous compromise are rarely discussed. Additionally, peroneal nerve injury occurs in approximately 25% of KDs, with surgical manipulation increasing this risk further. Our method of releasing and tagging this nerve was important in mitigating this risk [2,4].

At the conclusion of surgery, the knee was stable on varus/valgus stress testing as well as Lachman and posterior drawer assessments. Intraoperative fluoroscopy confirmed anatomic alignment, and peroneal nerve function was intact. Although long-term outcomes in KD-V injuries remain guarded, with arthrofibrosis rates reported up to 57% and post-traumatic arthritis even more common, our patient demonstrated early postoperative stability and preserved function. Rehabilitation remains individualized, with gradual weight-bearing and range-of-motion protocols tailored to fracture healing and ligament repair [10].

## Conclusions

This case demonstrates that complex KD-V KDs with bicondylar tibial plateau fractures can be effectively managed in a single-stage procedure through careful hemostasis, combined arthroscopic and open visualization, and precise surgical technique. Conservative treatment of the nondisplaced tibial plateau fracture and non-repair of the PCL minimized tissue trauma, scarring, and potential blood loss in the absence of a tourniquet, promoting better functional recovery. Accurate imaging is critical in diagnosing KDs, as spontaneous reduction can obscure the extent of injury and delay appropriate management. Additionally, acute compartment syndrome was successfully avoided through methodical surgical expertise in steering clear of the posterior compartment, preventing limb-threatening injury. The clinical course of this case emphasizes the need for caution, detailed imaging, and the establishment of standardized criteria to guide surgical decision-making, particularly when selecting between repair or reconstruction with an allograft versus autograft and between single-stage versus staged approaches for ligamentous repair.

By documenting our technical strategy, this report provides practical guidance for surgeons managing complex KDs in thrombosis-risk patients and contributes toward standardizing approaches to KD-V injuries. As this report describes only one case with short-term follow-up, the limitations in generalizability should be recognized, and the outcomes should be interpreted cautiously. Future efforts should prioritize reporting additional rare cases like this one to identify unique surgical considerations, better define critical timelines and operative strategies, and ultimately ensure optimal patient outcomes.
